# Observations on Solitary Versus Multiple Isolated Pancreatic Metastases of Renal Cell Carcinoma: Another Indication of a Seed and Soil Mechanism?

**DOI:** 10.3390/cancers11091379

**Published:** 2019-09-17

**Authors:** Franz Sellner

**Affiliations:** Surgical Department, Kaiser-Franz-Josef-Hospital, 1100 Wien, Austria; sellner.franz@aon.at

**Keywords:** renal cell carcinoma, pancreatic metastasis, seed and soil mechanism

## Abstract

Isolated pancreas metastases are a rare type of metastasis of renal cell carcinoma, characterized by the presence of pancreatic metastases, while all other organs remain unaffected. In a previous study, we determined arguments from the literature which (a) indicate a systemic–haematogenic metastasis route (uniform distribution of the metastases across the pancreas and independence of the metastatic localization in the pancreas of the side of the renal carcinoma); and (b) postulate a high impact of a seed and soil mechanism (SSM) on isolated pancreatic metastasis of renal cell carcinoma (isPM) as an explanation for exclusive pancreatic metastases, despite a systemic haematogenous tumor cell embolization. The objective of the study presented was to search for further arguments in favor of an SSM with isPM. For that purpose, the factor’s histology, grading, and singular/multiple pancreas metastases were analyzed on the basis of 814 observations published up to 2018. While histology and grading allowed for no conclusions regarding the importance of an SSM, the comparison of singular/multiple pancreas metastases produced arguments in favor of an SSM: 1. The multiple pancreas metastases observed in 38.1% prove that multiple tumor cell embolisms occur with isPM, the exclusive “maturation” of which in the pancreas requires an SSM; 2. The survival rates (SVR), which are consistent with singular and multiple pancreas metastases (despite the higher total tumor load with the latter), prove that the metastasized tumor cells are not able to survive in all other organs because of an SSM, which results in identical SVR when the pancreatic foci are treated adequately.

## 1. Introduction

Two fundamental mechanisms determine and regulate the metastatic potential of solid carcinomas. On the one hand, it is the imperatively necessary invasion of blood and lymph vessels by tumor cells: there can be no metastasis without circulating tumor cells or tumor cell clusters. The fate of the embolized tumor cells is just as significant, however, since clinical experience shows that not every tumor cell embolism inevitably results in clinically manifest metastasis. That is rather the result of a multi-stage interaction of tumor cells and host organ (seed and soil), since neither each tumor cell nor each host organ can provide all necessary properties required for successful interaction. Large-scale studies determined the complexity of this mechanism, which takes place in five main steps [1,2,3]: 1. The invasion of tumor cells in the vascular system “intravasion”; 2. The transport of the tumor cells in the vascular system; 3. The docking of the tumor cells, mechanically or by way of organ–tumor cell-specific adhesion to the vascular wall [1,2,3,4,5,6]; 4. The extravasion of tumor cells through the capillary wall in the extracellular matrix (ECM)—after distraction or death of endothelial cells [2,3,6,7,8,9,10,11,12], and 5. After previous dormancy [3,13,14,15,16] or without it, the maturation of the cells into metastasis (colonization); a process which requires the formation of a vessel network [17] and the overwhelming of the immune system of the host (interaction with ECM components [18] and stroma cells [2,19,20]).

In the light of recent studies, a pre-stage “0” still has to be added: the influence of the primary tumor on potential target organs, which takes place a long time before tumor cell embolization in order to prepare them for the subsequent metastatic settlement: the development of a pre-metastatic niche [3].

Paget recognized the essential significance of an interaction between circulating tumor cells and host organ in 1889 and concisely termed it the seed and soil mechanism (SSM) [21]. The main argument for his thoughts was the clinical observation that the individual malignant tumors do not spread the metastases diffusely in all host organs following an even distribution of the tumor cells with the bloodstream, but apparently have points of predilection (e.g., breast carcinoma and bone metastases). The second argument for a seed and soil mechanism is the relative resistance of individual organs and organ systems, respectively, to metastases, such as muscles or spleen. This behavior gives rise to the suspicion that local factors which can prevent the attack of metastases take effect in these organs [5]. Another clinical phenomenon could only be added to these two main arguments in favor of the effectiveness of an SSM in human medicine decades later; the absence of diffuse lung metastases after the placement of a peritoneo-venous shunt in the treatment of malignant ascites [22,23].

For that reason, it was all the more surprising that two studies [24,25] delivered substantial evidence indicating that, with isolated pancreas metastases in renal cell carcinoma (isPM), another clinical entity exists, the development and progression of which can be explained by an SSM.

The assumed great significance of an SSM with isPM [25] was based on the sole chain of proof presuming that the epidemiological studies favored a systemic-haematogenic metastasis route. That again calls for an SSM with isPM, since the exclusive growth of metastases in the pancreas despite a diffuse haematogenous tumor cell spreading requires a tumor cell selection. The objective of the study presented here was therefore to pursue the question as to whether no other unusual behavior patterns could be discovered among the numerous parameters presented for the characterization of isPM, which deliver arguments in favor of an SSM.

## 2. Results

### 2.1. History and Literature Compilation

The first description of a singular isPM is from Jenssen [26] from the year 1952; multiple pancreatic metastases in isPM patients were reported for the first time in 1984 [27,28]. In the time following, isPM were observed in very rare cases. Up until 1996, only 66 cases could be found in the literature [29]. That only changed with the progress that was made in pancreas diagnostics, which allowed the more frequent diagnosing of these tumors: In 2006, our working group was able to find as many as 239 reports [24], and 666 by 2016 [25]. By the end of 2018, 148 additional reports were added, which increased the total to 814 reports [24,26,27,28,29,30,31,32,33,34,35,36,37,38,39,40,41,42,43,44,45,46,47,48,49,50,51,52,53,54,55,56,57,58,59,60,61,62,63,64,65,66,67,68,69,70,71,72,73,74,75,76,77,78,79,80,81,82,83,84,85,86,87,88,89,90,91,92,93,94,95,96,97,98,99,100,101,102,103,104,105,106,107,108,109,110,111,112,113,114,115,116,117,118,119,120,121,122,123,124,125,126,127,128,129,130,131,132,133,134,135,136,137,138,139,140,141,142,143,144,145,146,147,148,149,150,151,152,153,154,155,156,157,158,159,160,161,162,163,164,165,166,167,168,169,170,171,172,173,174,175,176,177,178,179,180,181,182,183,184,185,186,187,188,189,190,191,192,193,194,195] (440 case records and 374 cases from summaries), which were used for the search for arguments in favor of an SSM.

### 2.2. Epidemiology and Pathology of isPM

The results of the literature analysis of the casuistic reports are presented in Table 1 [24,26,27,28,29,30,31,32,33,34,35,36,37,38,39,40,41,42,43,44,45,46,47,48,49,50,51,52,53,54,55,56,57,58,59,60,61,62,63,64,65,66,67,68,69,70,71,72,73,74,75,76,77,78,79,80,81,82,83,84,85,86,87,88,89,90,91,92,93,94,95,96,97,98,99,100,101,102,103,104,105,106,107,108,109,110,111,112,113,114,115,116,117,118,119,120,121,122,123,124,125,126,127,128,129,130,131,132,133,134,135,136,137,138,139,140,141,142,143,144,145,146,147,148,149,150,151,152,153,154,155,156,157,158,159,160,161,162,163,164,165,166,167,168,169,170,171,172,173,174,175,176,177,178,179,180,181,182,183,184,185,186,187,188,189,190,191,192,193,194,195].

Taken together, it produced the following results: 1. mean age 63.1 years; 2. 46% female, 54% male; 3. metachronous metastases in 93%; 4. interval from tumornephrectomy to manifest pancreatic metastasis 10.1 years (maximum 33 years [104]; 5. multiple metastases 38.1%; 6. Localization: head of pancreas 48%, body 22%, and tail 30%; 7. Grading: G1 16%, 2 64%, G3 20%, G4 0%; 8. cumulative five year survival rate (SVR) 72%.

#### 2.2.1. Histology

Since a standardized histological WHO classification gained acceptance only late in the observation period of over 65 years, only the cases of the last 20 years were analyzed. According to that, isPM are not strictly limited to clear cell renal carcinoma, but were occasionally also reported with the rarer renal carcinoma types: papillary renal carcinoma (*N* = 3) [172] and chromophobe carcinoma (*N* = 1) [172]. For that reason, an exclusive preference for one histological type for the occurrence of isPM cannot be deduced from the reports in hand, even though a frequent occurrence of isPM with clear cell renal carcinoma with 96% [172,178] is calculated on the basis of the reports up until now.

#### 2.2.2. Grading

The problem of the observation, the description, and the classification of individual tumor parameters varying across a long period of observation is also true for the grading of isPM. This is aggravated by the fact that a modification of the grading system for the WHO/International Society of Urological Pathology (ISUP) system [196,197] as carried out in 2013. In order to obtain comparable collectives, only those reports for which the classification according to Fuhrman which has been used most frequently in the past years were accepted for the analysis [198]. That was the case with only 137 cases reported in literature. The result of this analysis is shown in Table 1. There are 22 G1 cases compared with 88 G2, 27 G3 cases, and no G4 cases [172,175,183,186] (an undoubtedly G4 case was only reported for the first time in 2019 after completion of the literature research at the end of 2018 [199]).

#### 2.2.3. Singular-Multiple Pancreas Metastases

Since out of 456 sufficiently documented cases (322 case reports and 134 cases in summaries), at least 174 cases (38.1%) were described where multiple metastases developed in the pancreas from the beginning (case reports: *N* = 127 [24,26,39,44,45,50,52,55,57,60,62,65,67,70,74,79,81,82,86,88,90,92,93,96,97,99,101,102,104,105,106,107,112,113,115,119,120,124,127,131,133,139,141,144,145,146,147,153,158,159,163,166,170,171,182,188,190]; summaries: *N* = 47 [149,152,155,168,172,175]), this allows for a comparative examination of the multiple with the singular cases.

**Figure 1:**
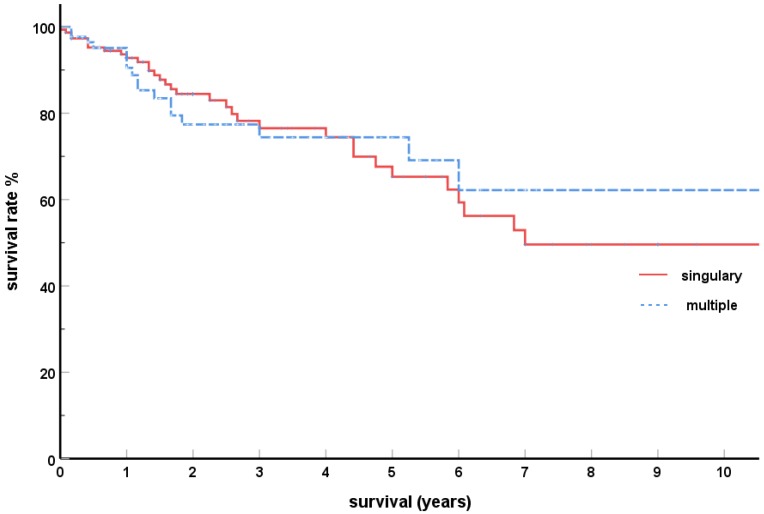
Kaplan–Meier survival curves; solitary versus multiple isolated pancreatic metastases from renal cell carcinoma (*p* = 0.557).

An average number of 3.1 (SD 1.5) pancreatic foci was determined based on the multiple reports, with up to 7 foci being reported [120,147], and even 14 in an individual case [168]. The results of the comparative analysis (Table 2) show that the two collectives of singular and multiple metastases do not differ significantly with regard to age, frequency of synchronous/metachronous metastases, interval until development of metastases, distribution of the grading levels, and cumulative 5-year SVR (Figure 1).

That is not surprising for factors such as age, synchronous/metachronous, and interval. But this behavior is unexpected and in need of explanation for the parameters of tumor grading and even more for the SVR.

The results of this literature analysis are already confirmed, at least regarding the SVR, by some single-institution reports which analyzed large collectives (*N* ≥ 20) by risk factors for survival; the four studies [152,172,174,175] confirmed that the presence of singular or multiple pancreas metastases has no significant impact on the SVR.

### 2.3. Arguments for a SSM in isPM

#### 2.3.1. Metastasis Route and SSM

Since the results in that regard were already previously presented in detail [24,25], the results updated with the new included cases [130,134,143,153,159,165,167,169,170,173,176,182,184,185,186,187,188,189,190,191,192,193,194] are listed here in the form of a summary.

Three potential metastasis routes are specified and discussed in the literature [25]:

1. A local lymphogenic metastasis route [48,88,101,106,150,161,168], where pancreatic metastases are supposed to develop by way of pre-existent or tumor-induced local lymph routes between kidney and pancreas after blockage of the regional lymph nodes by a retrograde lymphatic flow.
2. A local venous spread route, where pre-existent, porto-renal anastomoses [106,157,200,201], or draining, collateral veins of hyper-vascularized tumors [27,48,88,101,106,168]—enabling a tumor cell embolism in the pancreas—and that independently of whether there is a renal vein thrombosis or not [48].
3. The systemic haematogenic metastasis route

An analysis of the literature data produces the following results relevant for the issue of the metastasis route:

(a) Distribution inside the pancreas (Table 3)

Out of 210 sufficiently documented reported cases, 100 (48%) were described in the head, and 110 in the body and the tail area (52%). When that observed distribution is brought in relation to the volume distribution of the pancreas (46% in the head and 54% in the body and the tail [202]), no preference for one side can be found (*p* = 0.344). The distribution of isPM in the pancreas only correlates with the volume distribution and, consequently, with the blood flow.

(b) Distribution in the pancreas and renal carcinoma side (Table 4)

The distribution pattern of metastases inside the pancreas (head, body, or tail) indicates no dependency on left- or right-side renal carcinoma (*p* = 0.797), a result which was pointed out by our research as early as 2006 [24], and is now also confirmed by the collective of the casuistic reports published until 2018, and which is meanwhile also confirmed by several single institution reports [150,152,174,175,193].

Both results contradict a high significance of a local metastasis route and, in contrast, support the systemic haematogenic metastasis route. After all, the local-lymphatic route, as well as the local-venous route, should more frequently result in metastases in the nearby head of the pancreas after a right-side renal carcinoma and more frequently in the nearer body and tail area after a left-side renal carcinoma, from which a dependency of the distribution pattern in the pancreas from the location of the primary tumor should result. However, the contrary is the case. The detectable uniform distribution inside the pancreas—and especially its independence from the side of the primary tumor—can only be plausibly explained by a systemic-haematogenous metastatic pathway [24,25,88,147,161,168], since the risk of the development of metastases in all parts of the pancreas is equally high only there.

In addition to this main argument, we found more indications in the literature which argue against a high significance of the local-lymphatic and local-venous metastasis route, but in favor of the systemic route:

a. In general, the lymphatic system is rarely involved with isPM. Regional lymph node metastases were only present at the time of renal carcinoma surgery in 7.1% of the reports communicated [74,150,160,175,176], and at the time of pancreas metastases surgery, paraaortal lymph nodes were reported only once [142], and peripancreatic lymph nodes were found in only 18 of 309 reports (5.8%) [124,152,163,172,174,177]. That makes a great significance of a lymphatic tumor cell transport with isPM seem unlikely. b. A tumorous infiltration of the renal vein stems (category IIIb), condition for a flow reversal in the direction of the pancreas, was only reported in 9.6% [129,145,148,150], which argues against a great significance of tumor infiltration or tumor occlusion of the renal veins, respectively, in the development of isPM. c. Porto-renal anastomoses [200,201] which drain blood from the kidney region in the portal vascular system of the pancreas would, following the hepatopedal bloodstream in the portal vein system, also have to drain this blood with the tumor cells contained in it in the liver—with subsequent liver metastases. That is not the case, since no clustering of metachronous liver metastases is detectable epidemiologically. d. A high significance of a systemic-haematogenic metastatic route, however, emphasises that out of 27 extra-pancreatic, resectable metastases which developed time-wise between renal carcinoma surgery and diagnosis of pancreas metastases, 20 (72%) were undoubtedly of a haematogenic, systemic origin [25,153], and out of the 94 metastases which developed after the resection of the pancreas metastases, 70 (74.1%) were also undoubtedly of a haematogenic-systemic origin [25,130,167,169,170]. Consequently, the predominant number of metastases which were occasionally observed before the development or after the removal of pancreatic metastases are of a systemic haematogenic origin.

In sum, these data make a particular significance of the lymphogenic and local-venous route appear unlikely. By contrast, the systemic-haematogenic route seems to be more significant, since it correlates well with the epidemiological data. This comes, however, at the price that the question as to why—despite systemic-haematogenic spread—clinical manifest metastases develop exclusively in the pancreas remains unanswered [148,154,161,175].

#### 2.3.2. Histology, Grading, and SSM

The study found two relevant peculiarities. On the one hand, the highly specific metastatic pathway is not strictly linked to clear cell renal carcinoma, but was also occasionally observed with the rarer, histological forms [172]. On the other hand, the study shows that this highly specific metastatic behavior of the tumor cells gets lost with the degree of tumor cell de-differentiation; until the end of 2018, there was no G4 observation [172,175,183,186].

Particularities which indicate a special importance of an SSM cannot be deduced from the literature data for histology and grading.

#### 2.3.3. Multiple Pancreas Metastases and SSM

The comparative study of singular and multiple pancreas metastases presented here produced three results which are remarkable for the influence of an SSM:

1. The risk of multiple metastases in the pancreas, which is only 120–180 g, is high at 38.1%—with a simultaneous absence of metastases in other organs.

It shows that multiple tumor cell embolisms undoubtedly occur in the case of isPM. If manifested metastases remain limited to the pancreas despite multiple cell embolisms in the vascular system, however, that can only be explained by an SSM.

2. No different SVR for singular and multiple isPM.

That the SVR with metastasising renal cell carcinoma is influenced by the total tumor mass and therefore also by the number of metastases is to be expected, but it was only examined later in studies. When an effective drug treatment for metastasising renal cell carcinoma became available for the first time with the establishment of targeted therapies [169,203,204,205,206], the question as to prognostic factors gained clinical importance and resulted in corresponding studies. These studies [207,208,209,210], which differ little with regard to patient selection, determination of the tumor burden, and drug treatment show concordantly that the baseline tumor burden correlates significantly negatively with the overall survival time. Regarding the question asked here, this means that shorter SVR are to be expected for patients with multiple pancreas metastases and, consequently, a greater tumor load. However, the literature analysis presented shows—just like five major institution reports [150,152,172,174,175]—a diametrically opposite behavior; singularity or multiplicity of pancreatic metastases has no influence on the SVR.

3. A grading level distribution identical for singular and multiple metastases.

It is a known fact that the risk of tumor cell embolization and the subsequent number of metastases is co-determined by the degree of cell degeneration, i.e., the grading, in case of solid tumors, and that is the methodical reason for the grading of solid tumors. Regarding our question, however, this means that a shift to the higher grading levels should be detectable in the case of multiple metastases. However, the analysis revealed no such shift.

For that reason, the absence of the expected results for grading, and even more for SVR when comparing singular/multiple metastases, requires an explanation. However, the unusual clinical behavior can easily be reconciled with the effect of an exquisite SSM which is maintained over a longer period of time, often over several years. On the one hand, the embolised renal carcinoma cells with isPM have properties which make metastasis of them in the pancreas particularly easy, resulting in multiple metastases, while they cannot settle in the other organs on the other hand. Then, the high 38.1% rate of multiple pancreas metastases are not the consequence of more aggressive primary tumor cells with a larger number of embolized tumor cells, which cannot be reconciled with the non-different grading. The high rate is rather the consequence of a share of tumor cells able to settle in the pancreas which is exquisitely high with isPM. It is not the cell aggressiveness which determines the clinical course, but the exquisite cell adaptation to the pancreas, which is often maintained for years—consequently, a SSM. This SSM makes the non-different SVR plausible as well. In the case of singular as well as multiple metastases, the effect of the high organ specificity is that these cells are not able to metastasise outside of the pancreas and die. Since this mechanism affects tumor cells of singular metastases just like cells of multiple pancreatic metastases, a pattern of metastasis which is strictly limited to the pancreas persists for a prolonged period of time with both types of progression, which explains the consistent SVR after adequate treatment of the pancreatic foci.

## 3. Discussion

With over 330,000 cases world-wide, renal cell carcinoma is the ninth most common malignant tumor [211], which is already in a stage of generalisation in 20–30% of the patients at the time of diagnosis [171,172,175,211], and even after supposedly radical surgery, 15–25% of the patients develop a stage of generalisation [171,172,183,203] with metastases in lungs, bones, liver, and brain later on [157,178,183,212]. A special characteristic of renal carcinomas is that the disease is characterised by a protracted course in about 20%, with periods of slow tumor growth or stability for many years [144,171,181,212,213]. The exquisitely rare entity of isPM, of which approximatively 800 cases were reported world-wide, is also part of this last-mentioned group. The clinical presentation is typically characterised by a late onset (10 years after primary tumor), often multiple occurrence (38%), and a good prognosis. The only therapeutic option for a long period of time was surgical resection—depending on the metastatic site in the pancreas in the form of duodenopancreatectomy (DP) [26,116,148,171,175,186,193], pylorus preserving DP [120,121,127,131,132,135,137,140,150,174,179,183], distal pancreatectomy [29,116,145,157,181], total pancreatectomy [32,33,139,144,151,186,188], central (midsegment) pancreatectomy [68,121,131,146,147,152,171,175,186,193], or local tumor resections, the role of which is still controversial [25,121,125,146,150,152,158,160,163,172]. With cumulative 5-year survival rates of 43–100% (Table 5) (surgical treatment produced extraordinarily good results for metastasis surgery.

Over the last fifteen years, the medical treatment of metastatic renal cell carcinoma (RCC) has been revolutionized with the introduction of highly effective targeted therapies with multi-tyrosine kinase and mTor inhibitors, and monoclonal antibodies like angiogenesis and immune checkpoint inhibitors [154,169,203,204,205,206,214,215,216]. With these therapeutics, very positive results were achieved with isPM as well [160,178,217,218,219], which were even a match for surgical treatment in a retrospective study [178]. For that reason, the definition of the significance of surgical treatment, drug treatment, and the combination of both in isPM patients has to be the task of future prospective studies [154,160,175,179]. Only a few reports state that no anti-tumor treatment was carried out after the diagnosis “isPM”, and thus reflect the spontaneous progress, were reported. In 2018, a cumulative 3-year SVR of 42% [25] could be determined based on 16 cases. That was significantly worse than the SVR of the patients on whom curative resection was performed, but it is nevertheless an extraordinarily good result for an organ metastasis stage.

The generally good prognosis for patients with isPM clearly emphasises that this entity is not the accidental first manifestation of a directly upcoming stage of generalisation, but a separate, very special type of progression of metastasising renal cell carcinoma.

As the analysis of the literature overview shows, the reported epidemiological regularities (diffuse distribution inside the pancreas, no dependency on the side of the primary tumor, frequent development of undoubtedly haematogenic metastases in reports with extra-pancreatic metastases before or after the development of isPM) can ultimately only be rendered probable by way of a high significance of a systemic, haematogenic metastasis route.

However, the great significance of a systemic, haematogenic metastasis route with isPM deduced from the epidemiological data inevitably brings up the question of why, despite of a systemic haematogenous mode of cell spread manifest, metastases are strictly restricted to the pancreas. After all, the exclusive intravasion of the pancreas, which weighs only 120–180 g, by metastasised renal carcinoma cells by chance is highly unlikely, with the repeated cell embolisms in the scope of multiple metastases to boot. The only currently known mechanism which can explain that in a plausible manner is a SSM, which lets mature metastasising renal carcinoma cells into manifest metastases only in the pancreas, while they cannot develop to metastasis in all other organs.

With the theory of the local lymphogenic/haematogenic metastasis route as well—to which epidemiology assigns only little significance, however—isolated pancreas metastases can, with the simultaneously very rare presence of metastases in the complete tissue crossed by the tumor cells between renal tumor and pancreas (one paraaortal lymph node involvement [142]; only in 7% infiltrated peripancreatic lymph nodes [124,152,163,172,174,177]), also only be explained by a highly specific interaction between tumor cells and pancreas, which allows a maturation into metastases exclusively in the pancreas.

To date, the argumentation for the existence of an SSM for isPM was limited exclusively to conclusions drawn from the systemic-haematogenic metastasis route, which was rendered probable. The presented comparative studies of solitary and multiple metastases deliver a second chain of argumentation independent of the metastasis route for an SSM with isPM: the multiple pancreas metastases observed in 38%, the consistent grading level distribution for isPM with solitary and multiple metastases, and above all, the SVR, which are also consistent. That behavior can also only be plausibly explained by a highly specific SSM, which makes it much easier for these tumor cells to settle in the pancreas and makes it almost impossible in all other organs. Consequently, the disease remains strictly limited to the pancreas with solitary as well as multiple metastases, which results in equally good SVR with multiple as well as singular pancreatic metastases following an adequate “radical” treatment of the pancreas metastases (In context with the unusual clinical behavior of isPM it is noteworthy that a recent clinical investigation [220] revealed a second unexpected behavior of pancreatic metastases from renal cell carcinoma. In patients with multi-organ metastasis from RCC those seem to have an unexpected better outcome, in which the pancreas was affected from metastasis too, when compared with patients with multiorgan metastases but without pancreatic metastases—although the pancreatic metastasis group had a higher median number of affected organ sites than the non-pancreatic metastasis group. This indicates that RCC capable of metastasizing into the pancreas represent a special subgroup of RCC and that detailed molecular studies may provide valuable information on the molecular drivers of tumor progression [220]).

In summary, the study delivers another argument for considering isPM a model entity for the taking effect of an SSM, since the reports about singular and multiple metastases deliver an additional, second chain of argumentation. According to the current state of knowledge, several arguments consequently suggest that this type of metastasis should not exclusively be considered a mechanical tumor cell transport phenomenon, but to regard it as a biochemical tumor cell settlement phenomenon [3].

Limits of the study

It is a retrospective study of casuistic reports, and a hidden bias in the published casuistic reports cannot be excluded on principle. The methodical limitation of an analysis of casuistic reports is already compensated, however—at least as far as the SVR with singular/multiple metastases is concerned—by various high volume single institution reports [152,172,174,175] which confirm the results of the casuistic reports.

### 3.1. Pathomechanism

The precise biochemical processes involved in the development of isPM are not yet investigated and therefore unknown. Even in extensive overviews, detailed investigations concerning pancreatic metastasis of RCC could not be found [221]. There are, however, studies of more frequent tumor entities that identified some biochemical mechanisms that may also be at work in isPM.

#### 3.1.1. Genetic/Epigenetic Alterations and isPM

Due to the scarcity of isPM, detailed systematic studies on the genetic/epigenetic profile of isPM have, to our best knowledge, not yet been presented. A MEDLINE (PubMed) based literature research thus revealed only one single presentation [222], reporting on two cases of pancreatic metastasis in RCC in which mutation analysis was performed, in order to evaluate the use of moleculary targeted therapies. There are, however, a few publications comparing non-metastatic with metastatic RCC, however these report on metastatic RCC as one group without taking into consideration different distant metastasis sites or the inclusion of pancreatic metastasis. On the one hand, a recent investigation [223] did not address variability between matched primary tumors and metastasis or changes in the genomic of RCC. On the other hand, in a few publications [224,225,226,227,228], reporting on metastatic RCC as one group, it could be proved that the microRNA profile in metastatic RCC differs from non metastatic RCC [225,229], as well as the epithelial-mesenchymal transition associated microRNA/mRNA signature [230] (The number of differently expressed miRNA in metastatic RCC was determined to be 12 [229], 14 [224], 15 [230], 20 [227], and 21 [225], respectively; 11 of these miRNAs were mentioned just in one report: miRNA 10a–p5, 21, 30c–5p, 30e, 31, 130b, 149, 199–5p, 200b, 429, 455; and 10 in two or more reports: miRNA 10b–5p, 30a–3p, 30a–5p, 139–5p, 144, 200a, 200c, 204, 223–3p, 451). In addition, the microRNA expression in distant RCC metastasis differed according to the site of metastasis in lung, bone, and brain [224]. These results reveal a relation between microRNA signature and metastatic potential and distant metastatic site. Whether analogous a special microRNA signature is involved in the isPM phenomenon remains, of course, uncertain, and will have to be established by further investigations.

Furthermore, several investigations have shown the great number of altered miRNA involved in renal cell carcinoma also [228,231,232,233,234,235,236,237,238,239,240,241,242,243,244,245,246,247,248]. These miRNA regulate cancer metastasis also, because of their capacity to inhibit numerous target genes involved in different steps of the metastatic cascade, e.g., EMT [233,241,244,245], migration [234,236,238,240,241,246], and metastasis settlement [231,235,239,242,243]. The variable interactions of all these miRNS in various tumor cells brings about manifold different capabilities for metastasis, which increases the odds that one of the embolized tumor cell exactly “fits” the properties of the target organ (soil)—a necessary prerequisite for the metastatic process.

#### 3.1.2. Organotropism and SSM

The genetic mechanism responsible for this extreme organotropism of the metastatic pattern can naturally not be determined at present. In general, there will be an organ preference during metastasis if steps which require a precisely fitting interaction of tumor cell-specific properties with organ-specific properties occur in the scope of the early multi-stage process of metastasis. At least three such mechanisms are already known:

1. The pre-metastatic niche (PMN) [3,249,250,251,252]. PMN conceptualized as a fertile “soil” conducive to the survival and outgrowth of metastatic seed results from the interaction of three components: (a) Primary tumor derived components (such as tumor-derived secreted factors or tumor-derived exosomes/microvesicles [253,254]), (b) Tumor mobilized bone marrow derived cells (such as myeloid derived suppressor cells [255]), and (c) Organ components of the future host organ, (such as cellular and molecular factors, fibroblasts, endothelium cells of vascular structures, and extracellular matrix) [256,257]. Since properties of the primary tumor, as well as of the host organ, are involved in PMN formation, this results in an organotropism during niche formation, which facilitates later metastasis in the target organ and prevents it in non-target organs, respectively. The ability to provide a PMN is documented for RCC also, by the proof of a PMN in the lung [256]. This study also pointed out the complexity of the process, as only CD105+ tumor stem cells were capable of doing so. The ability to form a PMN in the pancreas, however, is so far not documented for RCC.

2. A successful interaction of a chemokine receptor on the tumor cell surface and a suitable ligand is the prerequisite for the activation of numerous signal transducing pathways, which are critical in cell proliferation, migration, angiogenesis, invasion, and proliferation [1,258]. Since the equipment of the individual tumor cells with chemokine receptors is tumor cell-specific and the type and the level of the ligand are organ-specific, successful interaction is only possible in tissues where cell receptors and ligand match precisely [1,2,3]. For example, breast cancer was found to express the chemokine receptors CXCR4 and CCR7 at high levels. The corresponding ligands on the other hand, CXCL12 and CCL21, are present at elevated levels in lymph nodes, lung, liver, and bone marrow—preferred distant metastatic sites of breast cancer [1,3].

3. Due to the lack of qualified investigations on pancreatic metastasis considerations on the impact of different immunoediting [259] in different distant metastasis sites, creating an organotropism leading to isPM must remain speculative, although conceivable, if one interprets the above-mentioned different miRNA profile at different distant metastatic organ sites as a consequence of cell selectioning process. It is therefore tempting to assume that in isPM in all host organs except the pancreas immune-surveillance detects and correctly eliminates the metastasized tumor cells by natural tumor specific T-cell mediated immune response, or keeps them in a dormant “equilibrium” state [259]. Only in the pancreas (soil) is an immunosuppression present which enables the carcinomas cells to evade immune control and to mature to manifest metastasis. IsPM would thus represent a “single organ deficiency of immune response”. The reasons for this immunosuppression in the pancreas are of course not yet known. In this context, it is worth recalling that the pancreas consists of two different components, an exocrine and an endocrine, and that at the moment it remains unknown whether the metastasis settlement commences in the vascular structures of the exocrine or endocrine component.

Currently, the question as to whether and how these biochemical processes, but also other phenomena still unknown to date, are involved in isPM remains unanswered, and has to remain the task of future research.

## 4. Materials and Methods

A literature compilation concerning isPM dating up to the end of 2018 was evaluated as to whether 1. The lines of argumentation presented so far are still tenable, and 2. Whether further epidemiological particularities and phenomena which can be explained by an SSM can be proven. Histology, grading, and a comparison of singular versus multiple metastases in isPM were examined and analyzed for correlations with the SSM for that purpose.

### 4.1. Data Sources

The literature research was based on MEDLINE (PubMed) registry and data on the epidemiology, pathology, and clinical variables i.e., age, sex, site of primary RCC, time to onset of metastatic disease, number, size, and site of metastases and survival time, were collected and analyzed.

### 4.2. Inclusion and Exclusion Criteria

For the analysis we considered as isPM the very rare observations of metastasising renal carcinoma where solitary or multiple metastases definitely, or at least across a period of years, synchronously, as well as metachronously to RCC, developed exclusively in the pancreas. For the selection of the individual reports, the criterions defined in 2006 (24) were used; e.g., for rating metastases as solitary or multiple, only those reports were considered that specified the number of lesions or that used wording clearly indicative of singularity or multiplicity. For defining the site of the metastatic lesions (head, body, or tail), only solitary isPMs that were unequivocally assignable to a specific part of the pancreas by preoperative imaging, the surgeons report, or the resected specimen were considered. It goes without saying that in a retrospective review, not every report contained data on all variables investigated, thus reducing the number of observations for subset analysis. The actual number of observations that provided information on a given variable was specified [24].

### 4.3. Statistics

Continuous data are presented as means (standard deviation). Differences were evaluated with the X^2^test, Fisher’s exact test, and Student’s t-test. Survival was calculated according to the Kaplan–Meier method and differences among subgroups were compared by log-rank test. *P* < 0.05 was considered significant.

## 5. Conclusions

IsPM constitute a very rare, well-defined tumor entity, for the development of which a highly developed SSM is responsible with a high probability. Furthermore, the uniform clinical course gives rise to the suspicion that the phenomenon is based on a uniform pathomechanism which remains constant for years. That indicates that genetic investigations would be meaningful to examine the mechanism which causes the exclusive presence of metastases in the pancreas and their absence in other organs, respectively. A clarification of these factors can contribute to a more profound understanding of the complex metastatic process, which is the fundamental requirement for the development of therapeutics which can block the metastatic process.

## Figures and Tables

**Table 1:** Analysis of 814 isolated pancreatic metastases from renal cell carcinoma [24,26,27,28,29,30,31,32,33,34,35,36,37,38,39,40,41,42,43,44,45,46,47,48,49,50,51,52,53,54,55,56,57,58,59,60,61,62,63,64,65,66,67,68,69,70,71,72,73,74,75,76,77,78,79,80,81,82,83,84,85,86,87,88,89,90,91,92,93,94,95,96,97,98,99,100,101,102,103,104,105,106,107,108,109,110,111,112,113,114,115,116,117,118,119,120,121,122,123,124,125,126,127,128,129,130,131,132,133,134,135,136,137,138,139,140,141,142,143,144,145,146,147,148,149,150,151,152,153,154,155,156,157,158,159,160,161,162,163,164,165,166,167,168,169,170,171,172,173,174,175,176,177,178,179,180,181,182,183,184,185,186,187,188,189,190,191,192,193,194,195]; *N* = number of cases with adequate documentation; (standard deviation of mean).

Variable	Data	%
Age (years; *N* = 349)	63.1 (9.7)	
Sex (m: f)	371: 318	54: 46
Synchronous: Metachronous	25: 334	7: 93
Time to Onset (years; *N* = 334)	10.1 (6.3)	
Multiple (*N* = 456)	174	38.1
Localization (head, body, tail)	99: 46: 61	48: 22: 30
Size (mm; *N* = 174)	37.0 (21.4)	
Radical Surgery (*N* = 477)	256	54
Grading 1, 2, 3, 4 (*N* = 137)	22: 88: 27: 0	16: 64: 20: 0
Actuarial 3-year Survival (*N* = 307)		80
Actuarial 5-year Survival (*N* = 307)		72

**Table 2:** Solitary vs. multiple isolated pancreatic metastases renal cell carcinoma (standard deviation of mean).

Variable	Solitary	Multiple	Significance
Age (years; *N* = 180/110)	63.7 (9.3)	62.7 (8.8)	n.s. *p* = 0.706
Synchronous	18 (11%)	6 (6%)	n.s. *p* = 0.432
Metachronous	153 (89%)	98 (94%)	
Time to Onset (years; *N* = 143/95)	10.1 (6.6)	10.0 (6.4)	n.s. *p* = 0.432
Grading (*N* = 27/23) 1	7 (24%)	4 (26%)	n.s. *p* = 0.670
2	11 (48%)	12 (41%)	
3	9 (28%)	7 (33%)	
4	0	0	

**Table 3:** Distribution of isPM within the pancreas. Right side = head, left side = body, and tail (*N* = 210).

Side Affected by Metasta	n	%
Right Side	100	47.6
Left Side	110	52.4

**Table 4:** Correlation between side of renal cell carcinoma and site of metastasis within the pancreas (*N* = 120; *p* = 0.797).

Site of Pancreatic Metastasis Site	Side Affected by Renal Cell Carcinoma		
	Left	Right	Bilateral
Head	36	28	1
Body	13	15	1
Tail	15	10	1
Total	64	53	3

**Table 5:** Cumulative 5-year survival rates following surgical treatment of isPM.

Author, Year	N	5-yr Survival (%)
Madkhali [193], 2018	17	47
Chatzizacharias [187], 2017	7	71
Fikatas [183], 2016	19	71
Yuasa [180], 2015	20	79
Tosoian [174], 2014	42	52
Schwarz [172], 2014	62	63
Kimura [168], 2014	13	77
Konstandinidis [152], 2010	20	61
Zerbi [146], 2008	23	88
Bahra [140], 2008	9	100
Crippa [132], 2006	5	80
Wente [131], 2005	12	53
Law [124], 2003	14	75
Bassi [121], 2003	22	53
Sohn [111], 2001	10	75
Thomson [104], 2000	21	43
